# The role of femoral offset and abductor lever arm in total hip arthroplasty

**DOI:** 10.1007/s10195-015-0358-7

**Published:** 2015-06-12

**Authors:** Filip Bjørdal, Kristian Bjørgul

**Affiliations:** University of Oslo, Hollandveien 26, 1555 Son, Norway; Østfold Hospital Trust, Aleris Health Oslo, Chr. Svendsens gate 6, 1771 Halden, Norway

**Keywords:** Hip arthroplasty, Minimally invasive hip arthroplasty, Femoral off-set, Uncemented, HOOS, Harris Hip Score

## Abstract

**Background:**

In order to create a well-functioning total hip arthroplasty (THA), it is important to restore femoral off-set and thus the abductor lever arm. The aim of this study was to investigate the clinical effect of increasing the abductor lever arm to and beyond the anatomical native lever arm in minimally invasive total hip arthroplasty performed through a direct anterior approach.

**Materials and methods:**

We compared the lever arm of the operated hip to the lever arm of the contralateral native hip on radiographs in 148 patients following THA. The patients were divided in two groups based on whether they kept their anatomical lever arm or had an increased lever arm. The clinical outcome was assessed using hip osteoarthritis outcome score (HOOS), Harris hip score and UCLA activity score.

**Results:**

Patients who kept their anatomical lever arm did not experience a significantly better clinical outcome than the patients with an increased abductor lever arm. We found no significant difference in clinical scores at any of the follow-ups during the first year after THA.

**Conclusion:**

The results of this study suggest that an increase in the abductor lever arm does not have major effects on the clinical outcome after THA. To avoid the potential negative effects of decreasing the lever arm, the surgeon should aim for an equal or slightly increased lever arm.

*Level of evidence* Level 3, prospective cohort study.

## Introduction

Total hip arthroplasty (THA) is a well-established treatment in patients suffering from arthritic disease of the hip, reducing pain and improving function [1]. By replacing the degenerative joint with a prosthetic stem and cup, one seeks to restore the normal anatomy of the joint, but several controversies remain regarding the optimal placement of the components [2–4].

In order to restore the optimal biomechanical forces of the joint, the acetabulum may be medialized, thus reducing the distance between the center of rotation and the body axis [5], which provides better mechanical conditions for the abductor muscles of the hip [6]. By medializing the cup there is a risk of reducing the global offset. It is therefore considered important to compensate with an equivalent increase in the femoral offset to ensure the biomechanical benefits [6–8]. However, when increasing the femoral offset, there is an inherent risk of exaggerated compensation, which may lead to increased tension on the abductor muscles and possibly pain and reduced function. We have not been able to find any literature investigating the clinical consequences of an overcorrection of the femoral offset.

The aim of this study was to investigate any correlation between a change in lever arm of the abductor muscles and clinical outcome, including the possible consequences of an exaggerated offset. To clarify this aspect we investigated whether there were any differences in clinical outcome between patients who had an increase in lever arm compared to patients who kept their anatomical lever arm.

## Materials and methods

During 2010 we performed THA in 166 patients using the direct anterior approach to the hip through the Smith-Petersen interval. Of these, 148 were included in our study group; 15 of the 166 patients were excluded due to previous contralateral hip arthroplasty, and 3 were excluded due to a decrease in the abductor lever arm (ALA) beyond 5 mm. All patients were followed and assessed with Harris hip score, UCLA activity score and hip disability and osteoarthritis outcome score (HOOS) with the added dimensions walking ability and recreational ability. Evaluations were made after 6 weeks, 4 months and 1 year postoperatively.

HOOS is a patient-administered questionnaire that consists of five subscales (pain, symptoms, activity of daily living, sport and recreation, function, and hip-related quality-of-life). Each question was answered using a Likert scale from 0 to 4 points and a score was calculated for each subscale, where 100 indicate no symptoms and 0 represents extreme symptoms [9].

The UCLA activity score is a scale ranging from 1 to 10, where 1 indicates inactivity and 10 the highest level of activity.

The THA was performed through the anterior approach on a fracture table. The method has been described thoroughly by several authors [10, 11]. All patients were mobilized on the day of surgery. We recommended partial weight bearing as needed and did not impose any restrictions on activities or range of motion. The implants used were an SL-PLUS MIA stem and a REFLECTION press fit cup (Smith and Nephew, Memphis, TN, US). The SL-PLUS MIA stem was available in a high offset version as well as in a normal offset version. In this study, we used exclusively the high offset stem, which has a CCD angle of 123°. The standard stem has an angle of 131°, and the difference in femoral offset between the stems is 8 mm when a size 6 stem is used with a neutral head.

### Radiological assessment

**Fig. 1:**
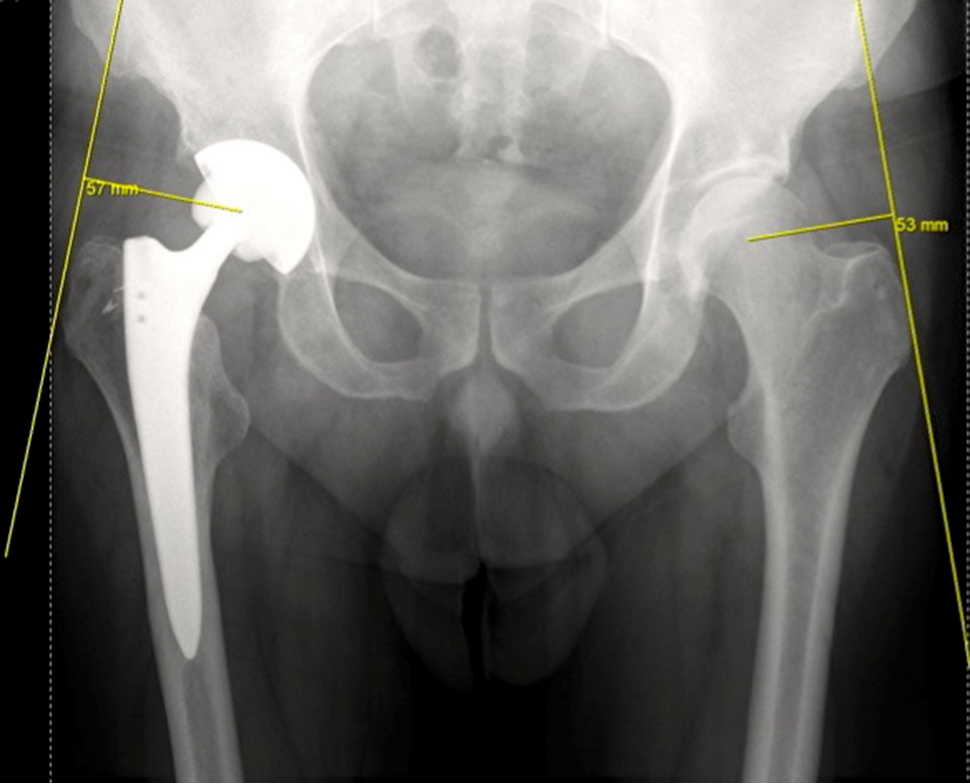
Radiograph demonstrating the abductor lever arm (ALA), defined as the distance from the center of rotation to the line of action of the abductor muscles

A standardized anteroposterior pelvic and hip radiograph was performed in all patients following THA. The ALA was defined as the distance from the center of the hip joint to the line of action of the abductor muscles (Fig. 1) [12]. The lever arm and the line of action of the abductor should form a 90° angle. The lever arm was then measured in the contralateral hip and compared to the operated side. The patients were divided into two groups based on the difference in ALA between the operated hip and the contralateral native hip. Group 1 consisted of patients with a lever arm restored to within 5 mm of the native lever arm, while group 2 comprised patients with a lever arm that was increased to greater than 5 mm of the native lever arm. The two groups were compared in regards to all parameters of Harris hip score and HOOS.

### Statistical analysis

Statistical analysis was performed using Microsoft Excel. Data were expressed as mean ± standard deviation (SD). Comparisons were made using the unpaired Student’s *t* test. A *P* value of less than 0.05 was considered to be significant.

## Results

The study population consisted of 51 men and 97 women with a mean age of 67.7 ± 10.9 years. Mean body mass index was 27.0 ± 4.3 (Table 1). An analysis of Harris Hip Score and HOOS preoperatively did not show any statistically significant differences between the two groups.

**Table 1:** Patient demographics

	Study population	Group 1	Group 2	*P* value
		ALA increase/decrease ≤5 mm	ALA increase >5 mm	
Number of patients	148	56	92	
Gender (male/female)	51/97	27/29	24/68	
Age (years)^a^	67.7 ± 10.9	66.2 ± 13.0	68.6 ± 9.3	0.23
Body mass index (kg/m^2^)^a^	27.0 ± 4.3	27.2 ± 4.5	26.8 ± 4.3	0.57
Preoperative clinical scoring				
Harris Hip Score	47.4 ± 18.1	46.4 ± 16.7	48.7 ± 18.6	0.45
HOOS––pain	35.7 ± 16.9	33.2 ± 16.0	37.3 ± 18.0	0.18
HOOS––symptom	40.6 ± 17.9	38.0 ± 18.1	42.2 ± 18.3	0.2
HOOS––ADL	36.7 ± 16.8	34.9 ± 17.3	38.2 ± 17.2	0.29
HOOS––sport/recreation	20.2 ± 18.9	17.9 ± 16.7	21.5 ± 20.6	0.28
HOOS––quality of life	27.8 ± 13.6	25.1 ± 11.9	29.1 ± 14.8	0.09
HOOS––activity 1a	2.7 ± 1.2	2.6 ± 1.0	2.7 ± 1.3	0.81
HOOS––activity 1b	2.6 ± 1.2	2.8 ± 1.2	2.6 ± 1.2	0.21
HOOS––activity 2	3.7 ± 2.0	3.5 ± 1.9	3.9 ± 2.0	0.19

*ALA* abductor lever arm, *HOOS* Hip disability and osteoarthritis outcome score

^a^Values are expressed as mean ± SD

### Radiological result

In our sample we found a native ALA of 58.0 ± 6.6 mm, whereas the mean lever arm of the operated side was 65.4 ± 5.9 mm.

**Fig. 2:**
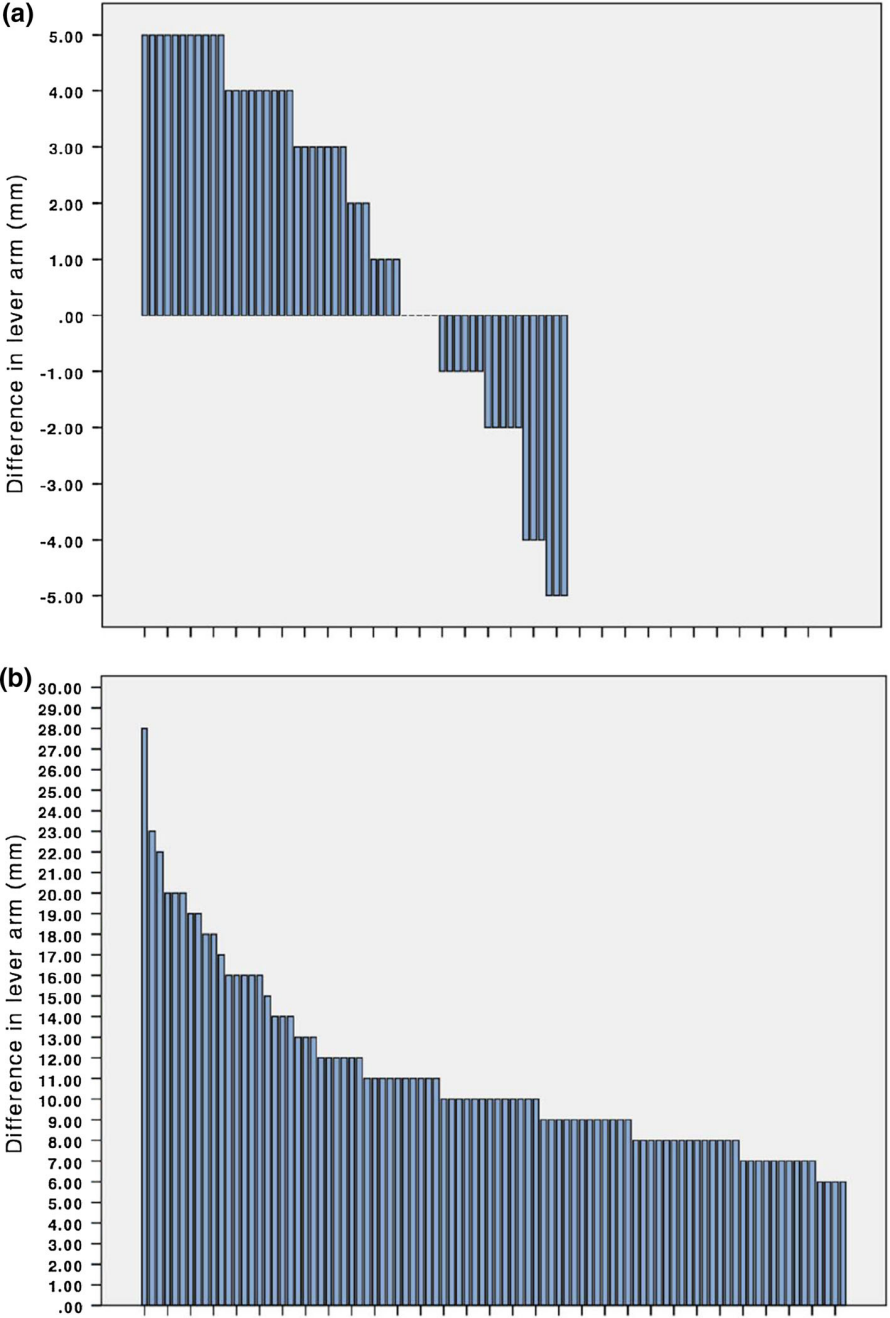
**a**, **b** Change in offset in patient groups 1 (**a**) and 2 (**b**). Each patient is represented by *one bar*

Group 1 consisted of 56 patients with a mean native ALA of 61.6 ± 6.1 mm. The mean lever arm of the operated side was 63.0 ± 5.4 mm; 17 of the patients in this group experienced a shortening of the lever arm, whereas 34 had an increase. Five patients did not experience a difference in lever arm between the two hips (Fig. 2a). The mean difference in lever arm between the contralateral native hip and the operated hip was 1.4 ± 3.12 mm.

Group 2 comprised 95 patients with a mean native lever arm of 55.8 ± 5.9 mm. The mean lever arm of the operated side was 66.9 ± 5.8 mm. These patients had a mean increase in the lever arm of 11.2 ± 4.3 mm (range 6–28 mm) (Fig. 2b).

### Clinical outcome

**Fig. 3:**
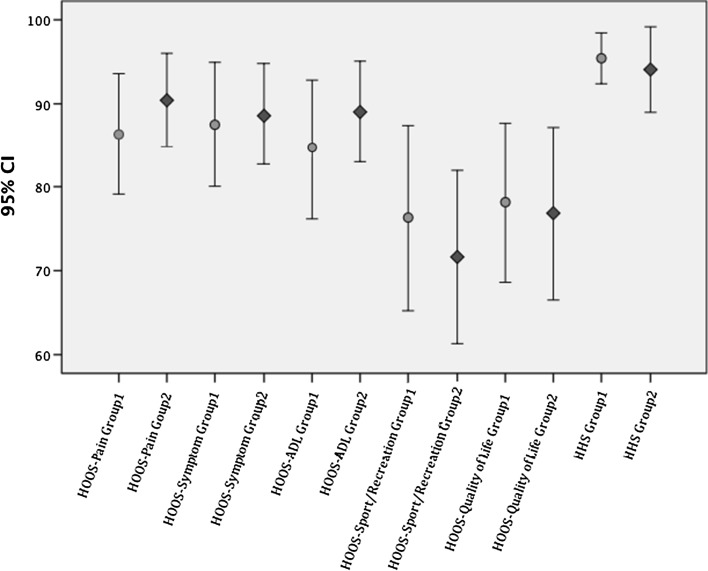
*Error bars* showing 95 % confidence intervals for the mean of hip disability and osteoarthritis outcome score (HOOS) subgroups and Harris hip score among group 1 (*circles*) and group 2 (*diamonds*). The two groups display overlap in all clinical parameters

Patients whose lever arm was restored to within 5 mm of the contralateral native hip did not experience a significantly better clinical outcome than the patients with a greater postoperative increase in lever arm (Fig. 3). After 1 year of follow-up there were still no statistically significant differences in any parameters of HOOS or Harris hip score between the two groups (Table 2).

**Table 2:** Clinical outcome 1 year after total hip arthroplasty (THA)

	Group 1	Group 2	*P* value
	ALA increase/decrease ≤5 mm^a^	ALA increase >5 mm^a^	
	Mean ± SD	Mean ± SD	
HOOS––pain	86.0 ± 19.0	91.3 ± 12.6	0.16
HOOS––symptom	86.7 ± 18.9	90.0 ± 13.0	0.37
HOOS––ADL	85.1 ± 20.1	87.8 ± 16.0	0.5
HOOS––sport/recreation	74.5 ± 27.5	73.4 ± 22.7	0.85
HOOS––quality of life	78.1 ± 25.0	79.4 ± 21.9	0.81
HOOS––activity 1a	3.7 ± 1.8	3.9 ± 1.5	0.69
HOOS––activity 1b	4.5 ± 1.5	4.2 ± 1.4	0.48
HOOS––activity 2	5.9 ± 2.4	5.8 ± 2.3	0.84
Harris hip score	94.1 ± 9.7	94.4 ± 10.6	0.86

^a^Values are expressed as mean ± standard deviation

## Discussion

Our data showed no significant difference in clinical outcome between the two groups at any of the follow-ups during the 1st year after operation. This suggests that a change in ALA does not have a large impact on the clinical outcome as measured by HOOS or Harris hip score during the 1st year after THA.

There is evidence that offset plays an important role when it comes to the clinical result following THA. Several studies have documented that an increase in offset results in increased range of motion, better mechanical advantage of the abductors and increased stability due to increased soft tissue tension [6, 12, 13]. Failure to restore offset has been associated with increased joint reactive force and hence an increase in polyethylene wear [14–16]. However, Little et al. [17] suggested that an increase beyond 5 mm of the contralateral hip might also result in increased polyethylene wear.

Although the importance of femoral offset in THA has been emphasized in several studies, there is limited research directly investigating the role of the abductor lever arm and its effect on clinical outcome. Studies have reported a correlation between the ALA and abductor muscle strength. McGrory et al. [12] reported that ALA length was among the most important factors influencing abductor muscle strength. Using a 3-dimensional biomechanical model, Delp et al. [8] demonstrated that lateral displacement of the hip center adversely affected the function of the abductor muscles by decreasing the lever arm, thereby decreasing the capacity to generate hip abduction moments. Recently, Terrier et al. [18] found that the benefits of cup medialization varies according to individual patient anatomy and stated that medialization should be balanced against possible disadvantages such as increased bone loss.

Our study provides clinical data that enables us to investigate how a change in lever arm affects the outcome after THA in a clinical setting where the surgery was performed by two surgeons using the direct anterior approach through the Smith-Petersen interval in every case. The same types of implants were used in all patients.

The radiological assessments were made using digital images from our database, enabling the radiologist to use measurement tools with high degree of precision. Furthermore, all measurements were performed by the same investigator (J.B.), which eliminated interobserver variability. Intraobserver variability was not assessed.

There are some limitations to our study. The patients were only followed for 1 year postoperatively. It is possible that more time is required to demonstrate a difference in clinical outcome. Another limitation may be that we did not perform an intra-observer validation study.

It is also possible that the instruments used to score the clinical outcome in our study lack sufficient sensitivity to demonstrate a significant difference between the groups. Although both HOOS and Harris hip score have shown a high degree of validity, it is possible that these instruments are not sensitive enough to demonstrate an underlying difference in clinical outcome between the groups [19, 20].

In our study population only 17 out of 148 patients experienced a shortening of the ALA. Several studies have reported that a shortening of the lever arm may result in weakness of the abductor muscles and reduced stability [6, 8, 12, 21]. It is possible that a higher frequency of patients with a decreased lever arm would have had a larger impact on the clinical scores.

The results of this study suggest that patients who preserve their anatomical ALA do not experience a significantly better clinical outcome than patients that have their lever arm increased. When considering the potential disadvantages of decreasing the lever arm, the surgeon should aim for an equal or slightly increased lever arm during THA.
